# Use of Artificial Genomes in Assessing Methods for Atypical Gene Detection

**DOI:** 10.1371/journal.pcbi.0010056

**Published:** 2005-11-11

**Authors:** Rajeev K Azad, Jeffrey G Lawrence

**Affiliations:** Department of Biological Sciences, University of Pittsburgh, Pittsburgh, Pennsylvania, United States of America; Georgia Institute of Technology, United States of America

## Abstract

Parametric methods for identifying laterally transferred genes exploit the directional mutational biases unique to each genome. Yet the development of new, more robust methods—as well as the evaluation and proper implementation of existing methods—relies on an arbitrary assessment of performance using real genomes, where the evolutionary histories of genes are not known. We have used the framework of a generalized hidden Markov model to create artificial genomes modeled after genuine genomes. To model a genome, “core” genes—those displaying patterns of mutational biases shared among large numbers of genes—are identified by a novel gene clustering approach based on the Akaike information criterion. Gene models derived from multiple “core” gene clusters are used to generate an artificial genome that models the properties of a genuine genome. Chimeric artificial genomes—representing those having experienced lateral gene transfer—were created by combining genes from multiple artificial genomes, and the performance of the parametric methods for identifying “atypical” genes was assessed directly. We found that a hidden Markov model that included multiple gene models, each trained on sets of genes representing the range of genotypic variability within a genome, could produce artificial genomes that mimicked the properties of genuine genomes. Moreover, different methods for detecting foreign genes performed differently—i.e., they had different sets of strengths and weaknesses—when identifying atypical genes within chimeric artificial genomes.

## Synopsis

Bacterial genomes contain genes that come from two sources; although most genes are inherited directly from parent cells at cell division, others may come into the genome from an unrelated organism. Often, these foreign genes can be detected because their sequences have compositional properties that differ from those of other genes in the genome. Methods for detecting atypical genes are difficult to assess because there are no genuine genomes wherein the histories of all genes are known. Here, the authors describe a method for creating artificial genomes that mimic the properties of genuine genomes, including containing “foreign” genes. The researchers used these constructs (a) to evaluate existing methods for finding foreign genes based on their atypical properties and (b) to test a new method for finding atypical genes. The researchers found that existing methods differ in their abilities to detect genes from different sources and that combining different methods can improve overall performance. The new method for finding atypical genes—which also identified sets of genes that share their unusual properties—worked very well in identifying potentially foreign genes in artificial, chimeric genomes.

## Introduction

With the number of genome sequences accumulating at a rapid pace, evidence for rampant lateral gene transfer among prokaryotes has increased dramatically [1−4]. Significant advances have been made in understanding this evolutionary phenomenon, and current research is aimed at understanding the impact of gene transfer rather than at demonstrating its occurrence [5−8]. Although inferences regarding the scope and impact of lateral gene transfer rely on the accurate and consistent identification of putative foreign genes, methods for objective, robust quantification of the lateral gene transfer have been difficult to devise. Unlike gene identification, where experimental validation of predictions is possible, it is difficult to ascertain the evolutionary history of a gene. In addition, there has been no platform available to test the efficacy and performance of methods for the identification of foreign genes. As a result, classification of genes as native or laterally transferred uses various sets of indirect evidence, and the scope and objectivity of each approach are debatable [9−13].

There are two primary strategies used to detect genes introduced by lateral gene transfer: parametric methods and phylogenetic approaches [3,14]. Phylogenetic methods detect putatively transferred genes by virtue of an unduly large degree of similarity among genes found in otherwise unrelated taxa and/or by the absence of orthologs in closely related taxa. The efficiency and reliability of this approach thus have a dependence on the depth and breadth of the sequence database and often rely on interpretation of discrepancies in relationships reflected by phylogenetic trees, themselves imperfect summaries of sets of relationships [15]. In contrast, parametric methods use the genome sequence of an organism to detect the genes that are atypical relative to the majority of genes in the genome; commonly used discriminant criteria include single nucleotide composition (SNC), dinucleotide composition (DNC), and codon usage bias (CUB).

While these two approaches are often used in concert to estimate the amount of genetic material transferred into a genome [3,14], parametric approaches are often invoked to assess whether particular genes may have been recently acquired because these analyses use only the information contained within the target genome and therefore do not require sister taxa for comparison. In addition, the results often appear to be more readily interpreted. Yet the efficacy of parametric methods lies in their ability to discriminate between typical and atypical genes, and to date no objective criteria have been offered to measure the robustness of parametric methods. This is due in part to the lack of genomes wherein the evolutionary histories of all genes are known with certainty.

As a result, critical issues remain relating to the discordant sets of atypical genes found by different methods for any species [9,10]. Both Ragan [9] and Lawrence and Ochman [14] speculated that different methods test different null hypotheses, thus leading to nonconvergent results. Moreover, each parametric method will necessarily balance the two types of classification error (failure to identify some foreign genes due to their similarity to native genes and misclassification of native genes as foreign due to some unusual character). This will lead to incongruent sets of putatively foreign genes being identified due to dissimilar thresholds for detection. Although these drawbacks could be alleviated by employing multiple identification methods and standardizing their classification error rates, the biases and error rates of most methods are not known.

Here we develop an approach to assess the abilities of parametric methods to detect atypical genes, thereby suggesting routes for establishing a unified approach for the identification of laterally transferred genes using multiple, complementary parametric approaches. To this end, we have developed a method for the creation of artificial, chimeric genomes using a generalized hidden Markov model (HMM) [16−19]. These artificial genomes reproduce the critical statistical properties of genuine genome sequences and therefore serve as valid test beds for evaluating both new and existing methods for the detection of laterally transferred genes. First, the genes composing the core of a genome—i.e., those genes likely not to have been introduced by lateral gene transfer and thus representing the spectrum of mutational signatures native to that genome—were obtained by using a novel gene clustering algorithm based on the Akaike information criterion (AIC) [20,21]; core genes were classified as “typical” by virtue of their nucleotide compositions, DNCs, and CUB patterns. Second, native genes were grouped using a *k*-means clustering algorithm that used relative entropy as a distance measure to decide the convergence of the algorithm [22]. Third, multiple gene models were derived according to these groups, so that artificial genomes could be generated by a generalized HMM using these gene models to represent the variability found among genuine “core” genes.

A set of artificial genomes modeled after genuine bacterial genomes was obtained. Chimeric genomes were generated as the mosaic collection of genes sampled randomly from different artificial genomes. Therefore, in these genomes, the evolutionary histories of genes as “native” or “transferred” were known with certainty. Using these artificial chimeric genomes, we tested the performance of several existing parametric methods for the detection of putative foreign genes, as well as novel methods for atypical gene identification based on the AIC. We discuss a framework for integrating multiple approaches, thereby allowing for more robust identification of foreign genes.

## Results

### Generating Artificial Genome Sequences

An artificial genome generator was constructed that produced protein-coding sequences and intergenic sequences using Markov models trained on genuine bacterial genome sequences. Protein-coding sequences were created by multiple, fifth-order, inhomogeneous Markov models; noncoding sequences were created by a homogeneous Markov model of noncoding sequence accounting for hexamer statistics. Separate models were derived for genes on leading and lagging strands. Structural RNAs, promoters, transcription terminators, and other features not commonly used in the identification of foreign genes were not included in genome models. The distributions of lengths of both coding and noncoding regions corresponded to those of the genome being modeled.

**Figure 1:**
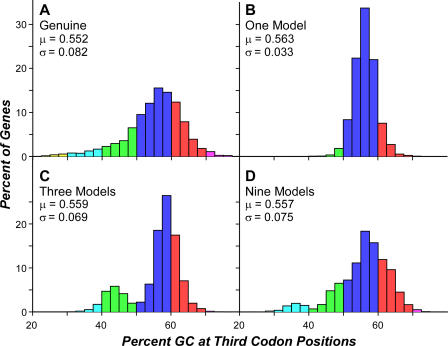
Variability within Genuine and Artificial *E. coli* Genomes Created with Variable Numbers of Gene Models The variability of percent GC at third-codon positions of genes is shown within the genuine *E. coli* genome (A), as well as artificial genomes created using one (B), three (C), and nine (D) gene models. Genes were clustered according to frame-specific DNC; μ and σ represent the mean and standard deviation of the distribution. For comparison between graphs, colors demarcate corresponding ranges of GC content.

All gene sequences in a bacterial genome cannot be accurately described by a single model; the probabilistic nature of the HMM would necessarily result in artificial genomes that failed to represent the variability among gene sequences seen in genuine genomes. For example, the genuine *Escherichia coli* genome contains far more variable genes than are contained in an artificial genome created with a single model accounting only for variability between genes encoded on the two DNA strands (Figure 1A and 1B). The spectrum of genes in genuine genomes results from numerous selective regimes acting upon genes in a single genome; e.g., genes experience a range of selection for CUB [23,24]. To resolve this problem, Markov models for protein-coding sequences were trained on sets of genes that reflected distinct directional mutational biases. To create appropriate training sets, genes within genuine genomes were grouped by their similarity in nucleotide composition, DNC, or CUB; segregation into distinct classes was achieved via the *k*-means clustering algorithm described by Hayes and Borodovsky [22] using relative entropy as a distance measure. As expected, artificial genomes generated by the HMM begin to recapitulate variability seen within genuine genomes when multiple gene models are used; e.g., if the *E. coli* genome was described by three or nine models, the resulting artificial genomes contained a more representative assortment of genes (Figure 1C and 1D) than did artificial genomes generated from a single gene model (Figure 1B).

### Optimizing the HMM for Generating a Genome Sequence

While increasing the numbers of models will allow the variability of genuine genomes to be more accurately represented, this tactic necessarily provides fewer genes in the training sets for each model. To optimize the HMM for number of gene models, we compared the distributions of nucleotide compositions and CUBs of genes within artificial genomes generated by the HMM to those in their genuine counterparts. As artificial genomes became more complex, the variability of such parameters among genes began to approximate that seen in their cognate genuine genomes. To measure the difference between artificial and genuine genomes, we calculated the cumulative χ^2^ of the differences of the three frame-specific percent GC distributions, using the distributions of these values in genuine genomes as the “expected” values. The cumulative χ^2^ values were plotted as a function of number of gene models; the minimum value in this curve was used to determine the minimal number of gene models required to encompass the directional mutational bias implicit in a genome.

**Figure 2:**
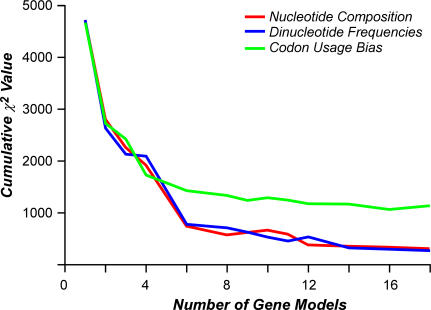
Goodness-of-Fit between Genuine and Artificial Genomes Created with Variable Numbers of Gene Models Genes within the genuine *E. coli* genome were clustered by nucleotide composition, frame-specific DNC, or CUB patterns. Correspondence between genuine and artificial genomes was calculated as the χ^2^ of the distributions of percent GC for the three-codon positions. Small χ^2^ values correspond to closer approximations.

Analysis of artificial *E. coli* genomes shows that the cumulative χ^2^ difference decreases sharply as the number of gene models increases until an optimum number of models is reached (Figure 2), after which increasing the number of gene models in the HMM did not result in any significant change. As very large numbers of gene models are used, the cumulative χ^2^ difference increases, as apportioning fewer numbers of genes into each model decreases the accuracy of the HMM. For the three discriminant criteria—SNC, DNC, and CUB—tested in the *k*-means clustering algorithm, variability of nucleotide composition within the *E. coli* genome can be quite closely approximated by using about 10–12 gene models (Figure 2). Closest approximation used somewhat larger numbers of gene models, but the improvement in fit was only marginal; optimal numbers for the artificial *E. coli* genome were 12, 14, and nine models for clusters formed using the SNC, DNC, and CUB criteria, respectively.

**Figure 3:**
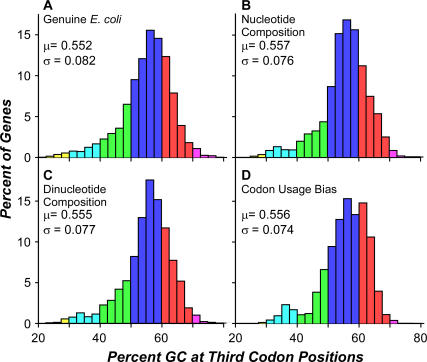
Variability within Genuine and Artificial *E. coli* Genomes Using Different Sets of Gene Models The distribution of percent GC of the third-codon positions of genes within the genuine *E. coli* genome (A), as well as artificial *E. coli* genome generated from *E. coli* genes clustered by SNC (B), DNC (C), or CUB (D). Artificial genomes were constructed using the optimal number of gene models (see Figure 2); μ and σ represent the mean and standard deviation of the distribution. For comparison between graphs, colors demarcate corresponding ranges of GC content.

**Figure 4:**
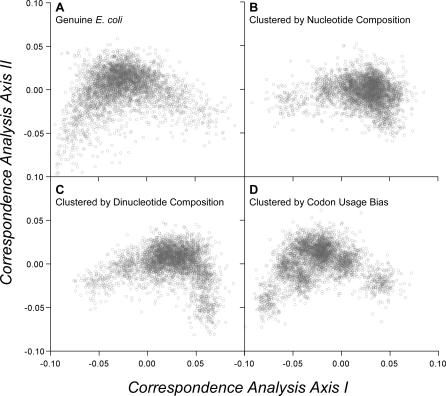
Correspondence Analysis of CUB The first axes—indicating variability in usage among 59 synonymous codons—are plotted for genuine *E. coli* genes (A) and genes from artificial genomes (see Figure 2) created from gene models sampling groups of genes clustered by SNC (B), DNC (C), or CUB (D) criteria.

In artificial genomes constructed with the optimum number of gene models, the variability in nucleotide composition at each codon position closely approximated that seen in the genuine *E. coli* genome; the plot for percent GC at third-codon positions is shown in Figure 3, although clustering using CUB criteria performed less well (see Figures 2 and 3D). To examine variability in CUB, we created factor maps from the first and second axes of correspondence analyses using software developed by McInerney [25]. In the plot for genuine *E. coli* genes (Figure 4A), the shape of the now-famous “rabbit head,” as first described by Médigue et al. [26], is evident. Here, the majority of *E. coli* genes share a similar CUB, highly expressed genes form one “ear,” and laterally transferred genes—bearing more unusual CUBs—form the other “ear.”

This shape is also apparent in the factor maps obtained for artificial genomes created from genes clustered by either the DNC and CUB criteria (Figure 4). However, this distribution is not evident for genes clustered by similarity in nucleotide composition (Figure 4B), indicating that CUB information is lost. That is, these sets contain genes with disparate CUBs, resulting in less-informative models. The factor map for the genome based on genes clustered by the CUB criterion also appears to be more fragmented (Figure 4D), likely because each model was trained on a set of genes with highly similar CUB profiles. These observations led us to conclude that the HMM with gene models derived after clustering genes using DNC as a discriminant criterion is most effective in modeling the mutational bias patterns specific to a prokaryotic organism. That is, it captures genic complexity in both nucleotide composition and structure.

### Extracting the Core Genes of a Genome

In developing artificial genomes for evaluating parametric methods for detecting atypical genes, we wished to create chimeric genomes with genes “donated” from different artificial genomes, each modeled after a different genuine genome. Yet most genuine genomes include both foreign and native genes [3,27], potentially confounding the training sets selected to represent the variability of native genes within genomes. Therefore, we must eliminate from the HMM training sets any atypical genes likely to have been recently introduced through lateral gene transfer. While the number of vertically inherited genes decreases as one compares genes that are more distantly related [27], the majority of genes in bacterial genomes have been resident there for sufficient time to acquire similar sequence characteristics [28−30]. That is, robust models representing the spectrum of native genic variation within a genome can be created if the most atypical genes are first excluded.

We identified genes likely corresponding to the native, vertically inherited “core” genome using a parametric clustering method based on the AIC (see Materials and Methods). As expected, the number of genes in the core genome depended on the criteria used to cluster them. For example, by applying the AIC gene clustering algorithm to a set of 4,255 *E. coli* protein-coding genes, 3,026, 2,643, and 3,031 genes were identified as native genes when using frame-specific single nucleotide bias, frame-specific dinucleotide bias, and CUB as discriminant criteria, respectively. Here we chose the set of 2,141 genes identified by all three criteria, representing the high-confidence set of core genes; this AIC-generated core was used for subsequent analyses.

Correspondence analysis of the core *E. coli* genome, similar to that shown in Figure 4, shows that the “ears” of the rabbit head—representing both atypical genes and highly expressed native genes—have disappeared (Figure S1). The removal of very highly expressed genes from the *E. coli* core genome is neither unexpected nor unwanted. Because highly conserved genes are both transferred less frequently [8] and more readily identifiable as “native” due to their readily identified functions, refining parametric methods to detect them is unnecessary. Therefore, the core genome represents a framework against which all atypical genes can be detected.

Aside from their sequence properties, the identities of genes included and excluded from the *E. coli* core follow predictable patterns. As expected, genes for “housekeeping” metabolism—those directing amino acid biosynthesis and central metabolism—were included in the core genome. Three classes of genes were noted to be excluded. First, mobile genetic elements (transposons and genes within prophages) were excluded, likely because of their unusual CUB. Second, other genes of known foreign origin, identified through either parametric analysis [31] or phylogenetic analysis [13], were also excluded (e.g., genes of the *phn, rhs, hsd, rfb,* and *lac* operons). Third, highly expressed genes—e.g., those encoding ribosomal proteins and elongation factors—were also excluded, as predicted from the correspondence analysis. Overall, the number of genes in the core genome is comparable to the number of protein-coding genes shared between *E. coli* and its sister taxon, *Salmonella,* that are greater than 300 nucleotides in length. These data indicate that this approach does provide a reasonable collection of genes that would reflect the major portion of the spectrum of native mutational biases. More important, it is against this variability that atypical genes must be detected; therefore, these genes represent ideal candidates for the construction of artificial genomes.

### Generating Artificial Core Genomes and Chimeric Genomes

The core genes of a bacterial genome were obtained as described above and were segregated into distinct classes by the *k*-means gene clustering algorithm using frame-specific DNC as the discriminant criterion. Given the performance of the HMM in representing the variability within complete genomes, we expected even better performance when the most atypical genes were excluded from the training sets. The number of gene models was selected using the optimization technique described above. The gene models derived from these clusters were used in the HMM to generate artificial core genomes reflecting the characteristics of the cognate genuine core genomes; the number of genes created by each gene model was proportional to the number of genes in its training set. As was the case when entire genomes were being modeled, parametric properties such as the frame-specific nucleotide composition (Figure S2) and CUB (see Figure S1) of genes in the artificial core genome reflect those of the genuine core genome being modeled. The variability of genes within the artificial core genome—reflecting the range of that seen in genuine core genomes—again justifies the use of HMM with multiple gene models.

To create artificial genomes that have experienced simulated lateral gene transfer events, the core genomes of several prokaryotic organisms were modeled by the genome generator; for each core genome, the optimum number of gene models was used. Chimeric genomes were then generated as mosaics of genes taken randomly from several synthetic genomes in predefined proportions. In this way, artificial genomes can be created with varying proportions of foreign genes from a large number of sources. More important, the history of genes in these artificial genomes—i.e., whether genes are “native” or “foreign”—is known with absolute certainty. Because each core genome is described by multiple gene models, several hundred gene models may be used to create even the most simplistic chimeric genome, thereby providing the high degree of variability among genes observed in genuine genomes.

### Evaluating Parametric Methods for Detecting Atypical Genes

Numerous chimeric genomes were generated and analyzed by the parametric methods to detect atypical genes (see Materials and Methods). We present here the results from analyses of mosaic artificial genomes containing 4,000 genes, with the majority (85%) generated from the *E. coli* core gene models. The “foreign” genes were modeled after core genomes derived from *Archaeoglobus fulgidus* (1%), *Bacillus subtilis* (1%)*, Deinococcus radiodurans* (2%), *Haemophilus influenzae* Rd (2%), *Methanococcus jannaschii* (1%), *Neisseria gonorrhoeae* (1%), *Ralstonia solanacearum* (2%), *Sinorhizobium meliloti* (2%), *Synechocystis* PCC6803 (1%), and *Thermotoga maritima* (2%). We implemented several methods to identify atypical genes; in this case, the artificial *E. coli* core—contributing 85% of the genome—was considered to be the recipient genome, and the ten other artificial genomes were considered to be donors for simulated lateral gene transfer events. To evaluate the performance of each method, two error rates were considered. Type I error (false negative) was calculated as 100 – sensitivity, where sensitivity is the percentage of foreign genes correctly identified as foreign. Type II error (false positive) was calculated as 100 – specificity, where specificity is the percentage of predicted foreign genes that were actual foreign, i.e., created by a model trained on non−*E. coli* genes.

**Figure 5:**
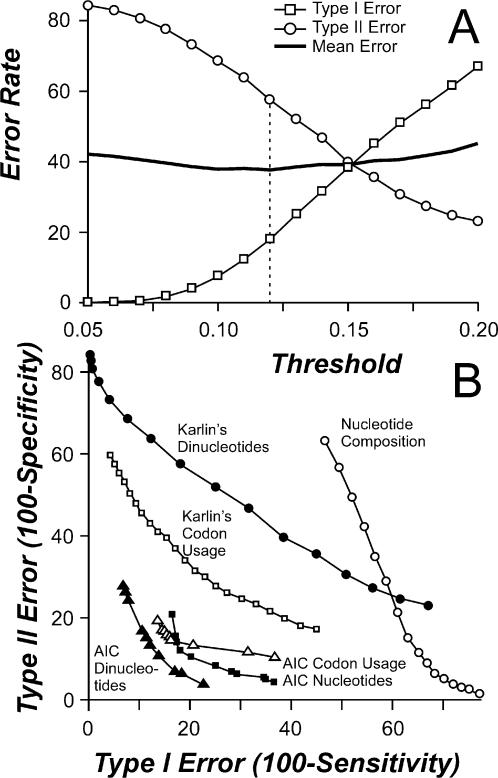
Tradeoffs in Error Rates in Methods for Detecting Atypical Genes (A) Type I error, type II error, and mean error for predicting foreign genes according to Karlin's DNC method [32]; the dashed line indicates the minimum mean error. (B) Tradeoffs in error rates for several methods of gene detection.

As expected, there was a tradeoff between type I and type II errors, i.e., as methods became more sensitive in detecting foreign genes (lower type I error), they were also less specific and misclassified more native genes as putatively foreign (higher type II error). As an example, Figure 5A shows the results for Karlin's dinucleotide method [32], where the threshold parameter determines which genes are considered sufficiently atypical to be deemed foreign. This tradeoff is seen for all methods examined (Figure 5B). As expected, more conservative thresholds result in lower type II error and higher type I error. The use of artificial genomes enables users of these algorithms to evaluate the stringency of their threshold criteria prior to application of these methods on genuine genome sequences. Alternatively, one could use the differential performance of the method to assign confidence values to atypical gene assignments, i.e., genes declared “foreign” at low threshold values would have higher confidence than those declared foreign at high threshold values, where type II error was greater. To compare the performance of different methods, we established optimal threshold criteria that minimized the average error rate (Figure 5A).

The performances of several methods for identifying foreign genes—each using threshold criteria that minimized their mean error rate—are compared in Table 1. Several results are notable. First, it is clear that the efficiency of detecting foreign genes depends on the source of the gene. For example, Karlin's codon usage method performed well in identifying genes from *A. fulgidus, R. solanacearum,* and *M. jannaschii* but comparatively poorly in identifying genes donated from *B. subtilis, N. gonorrhoeae,* or *Synechocystis* PCC6803 (Table 1). Second, sets of foreign genes detected well by some parametric methods were not detected as well by others. For example, Karlin's dinucleotide method did well in identifying foreign genes introduced from *Synechocystis* PCC6803 but not from *D. radiodurans;* Karlin's CUB method had the opposite tendency, performing poorly in identifying foreign genes from *Synechocystis* PCC6803 and doing fairly well with those from *D. radiodurans*. Third, it is clear that—at least in identifying genes from this test set—some methods are more robust than others; the average error rates showed substantial variation. Some methods minimized both type I and type II errors (visualized on Figure 5B as curves that approach the intersection of the axes) better than others. As a point of comparison, identifying foreign genes solely on the basis of atypical nucleotide composition can show very low type II error (indicating that few suspected foreign genes are actually native) but very high type I error (indicating that many foreign genes were not identified).

**Table 1:**
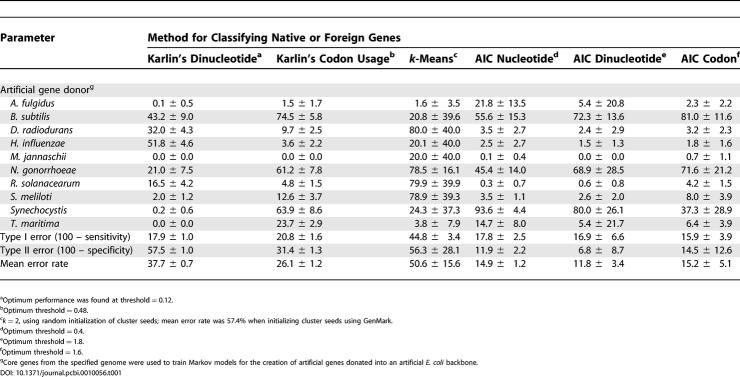
Error Rates of Parametric Methods for Detecting Atypical Genes in an Artificial *E. coli* Genome

### The *k*-Means Clustering Algorithm Fails to Identify Genes from Variable Sources

The *k*-means clustering algorithm has been implemented on genuine genomes to group genes into either two or three clusters, where one cluster is labeled as foreign [22]. When applied to chimeric artificial genomes, this method produced high values of both types of error for *k* = 2 (two clusters, Table 1). This result is not unexpected, because not all atypical genes are alike and would not be segregated into a single cluster. For *k* = 3, one of the three clusters contained predominantly (>95%) native genes and one cluster contained predominantly (>95%) foreign genes. The third cluster typically contained approximately 60% native genes, and assignment of this third gene cluster as either native or foreign would produce either a high type I or high type II error.

If the weakness of the *k*-means method lay in the high variability of foreign genes in artificial genomes, then reducing the complexity of the artificial genome should improve the performance of this method. Therefore, we constructed another set of artificial genomes with 75% *E. coli*−derived genes and the remaining genes from five other artificial genomes (modeled after *A. fulgidus, M. jannaschii, B. subtilis, R. solanacearum,* and *H. influenzae,* at 3%–6% abundance per genome). Using these less-complex genomes, the *k*-means clustering algorithm performed better, and the mean error of 13.0% compared favorably with the error rates of other methods (Table 2). In addition, while Hayes and Borodovsky [22] initiated their analyses using cluster seeds derived from the GenMark algorithm, we found that random cluster seeds were equally effective (Table 2). When the proportion of *E. coli* genes was increased to 85%, type II error remained the same and type I error increased slightly to 24.5% (data not shown). We conclude that when foreign genes are less diverse, the *k*-means method performs better. Similar improvements were not observed for other methods (Table 2), and the AIC-based approaches remained the most robust.

**Table 2:**
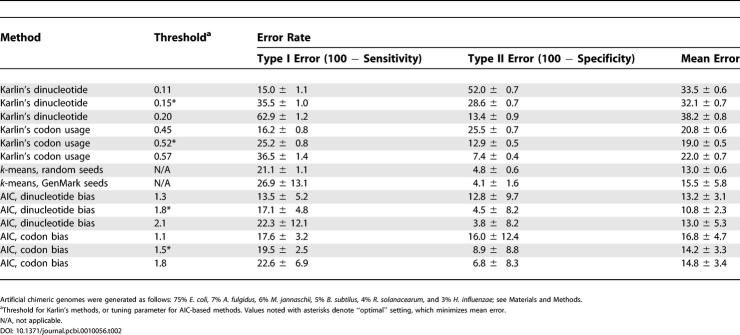
Error Rates of Parametric Methods for Detecting Atypical Genes in an Artificial *E. coli* Genome

### Using the AIC to Identify Atypical Genes

We used artificial genomes as a platform to test the implementation of a novel method for detecting foreign genes using the AIC [20]. Here, genes within chimeric, artificial genomes were clustered using either nucleotide composition, DNC, or CUB as the discriminant criterion (see Materials and Methods). Initially, genes were assigned to individual gene clusters (i.e., clusters containing a single gene). The pairwise distances between clusters were assessed using the AIC, and the closest clusters were merged if ΔAIC was negative, i.e., if the *N –* 1 cluster model better described the data than did the *N* cluster model. This process was repeated until cluster merger was no longer significant (see Materials and Methods).

The largest cluster was inferred to contain “native” genes, because native genes would be the most numerous genes in a genome; smaller clusters were inferred to contain foreign genes that failed to be merged with the primary cluster because of their atypical sequence features. This approach of assigning a single native gene cluster worked well for the analysis of artificial genomes, where unusual native genes have been excluded from the “core” genomes (see Figure S1). When applied to genuine genomes, additional clusters containing highly expressed genes would also be denoted native; this assignment should not be problematic or contentious, because the ancestry of these genes is rarely in doubt [33].

Two features of the AIC-based approach are salient. First, the number of clusters arrived upon by this method is not predetermined, as it is with the *k*-means algorithm [22]. Because the numbers and features of foreign genes cannot be predicted, the AIC-based clustering method avoids arbitrary assignment of genes into clusters. Second, clusters may contain a single gene if they were never merged with other gene clusters. In this way, foreign genes that are not similar to other genes are still identified as foreign. That is, the AIC clustering method does not derive a description of foreign genes and cluster them together; rather, typical genes are identified and grouped together, and foreign genes are those that do *not* fall into the cluster of native genes. Third, foreign genes that have similarity with each other *are* clustered, which serves as a form of validation. That is, groups of genes with suspected common foreign origin—e.g., the *E. coli phn* operon [34] or the *Salmonella cob* operon [35]—should fall into the same cluster.

The error rates produced by the new AIC-based gene clustering methods show that they perform very well, outperforming the other methods described (see Figure 5B; Tables 1 and 2). For example, in examining artificial genomes with laterally transferred genes from ten sources (Table 1), the mean error rates for the AIC-based methods (12%−15%) were far lower than Karlin's dinucleotide (37%–39%) or CUB (26%−28%) methods. Overall, the AIC clustering method using DNC performed the best on these data, minimizing both type I and type II errors (Figure 5B). Similar results are seen when analyzing the five-donor genome case (Table 2). In addition, the overall performance of this method did not rely heavily on the value of the “tuning” parameter (see Materials and Methods), which is analogous to threshold parameters of other methods. As seen in Table 2, all methods show a tradeoff between type I and type II errors; for the AIC-based methods, small adjustments in the tuning parameter did not dramatically alter performance. The performance of the AIC-based methods does not reflect the composition of the core genomes, which were generated via an AIC-based clustering algorithm. When core genomes extracted using Kullback-Leibler (K-L) distance were used to train Markov models used to generate artificial genomes, nearly identical results were obtained (Figure S3).

### Performance in Classifying the Short Open Reading Frames

**Figure 6:**
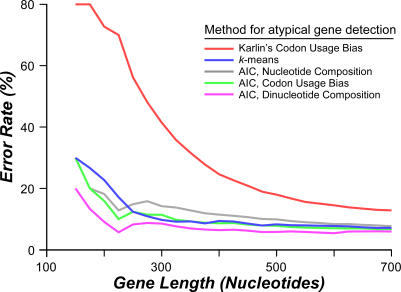
Performance of Parametric Methods in Classifying Short Genes The error rate in proper classification of genes as native or foreign as a function of gene length was assessed for genes within chimeric, artificial genomes.

Short open reading frames are commonly misclassified as putative foreign genes when parametric methods are applied [14]. Although short genes may encapsulate useful biological information in their structure, they may appear as noise in the statistical analysis. There must be a minimum length beyond which a gene fails to provide robust data for statistical analysis, but this threshold is not obvious; in many analyses, it has been arbitrarily set to 400 nucleotides [14]. In addition, different methods may have different sensitivities vis-à-vis short genes. We examined the performance of the methods used to detect atypical genes as a function of gene length (Figure 6). For most methods, one can easily conclude that genes in excess of 250 nucleotides can be easily classified; therefore, the threshold of 400 nucleotides is valid, although somewhat more conservative than is necessary. The exception to this trend is Karlin's CUB method, which performed poorly in classifying short genes but improved as gene length increased (Figure 6). This behavior was not solely the result of CUB providing insufficient information for identification of short genes; the AIC-based clustering method that uses CUB as a discriminant criterion performed well in identifying short, foreign genes.

## Discussion

### Artificial Genomes Provide a Useful Evaluation Platform

The performances of several methods were evaluated by a test system using chimeric artificial genomes, which has allowed us to critically analyze the limitations of parametric methods for detecting laterally transferred genes. These results provide to our knowledge the first comparative assessment of the abilities of parametric methods. The tradeoff between type I and type II errors has been evaluated, and differential performance in detecting genes from different source genomes has been demonstrated. In addition, methods using the same discrimination criterion—e.g., CUB implemented by both Karlin et al. [36] and the AIC-based method described here—have shown significantly different results, suggesting that alternative analytical approaches using similar data are worth pursuing.

Genomes are enormously complex sequences, and it would be fair to consider even domains of genes to represent sequences under unique selective constraints. In addition, genes are organized into operons and are regulated in complex networks; each level of complexity imparts characteristic details that could be modeled at the sequence level. Considering phylogenetic paradigms where the interactions are at the genome level, thus obviating the need to look at more obtrusive levels of complexity, genes that have evolved under similar conditions can be described by a distinct model. While simple models for artificial genome construction based on nucleotide or hexamer statistics (e.g.*,* GenRGenS [http://www.lri.fr/~denise/GenRGenS/]) are suitable for examination of regulatory interactions or performance of artificial life simulations [37,38], more sophisticated models are required to accurately assess the performance of algorithms in detecting atypical genes in genuine genomes.

We exploited the directional mutational bias driving genome evolution to optimize the HMM for a minimal number of gene models. The artificial genomes we constructed represent a simplification of the complexity underlying a genome. The factor maps for genes of artificial genomes show some discontinuity, representing the centers of clusters as compared to the continuous distribution observed for real genome (see Figure 4). The finite number of gene clusters used to train gene models does not reproduce the subtle complexities of bacterial genomes; rather, the gene clusters represent the major trends observed among the core genes. Some apparently unusual (atypical) genes left unfiltered by the core extraction method are thus not represented in an artificial genome. An artificial genome is intended to model certain characteristic variations among genes that are exploited in the detection of foreign genes. Other complexities of genome sequences were not modeled but could be included if they were deemed useful or important.

The performance of atypical gene identification methods could be examined with or without additional, more complex information included. For example, strand bias was included in our artificial genome generator, but artificial genomes can be generated that lack strand identity (see Figure S4); therefore, the sensitivity of methods to this aspect of genome complexity could be assayed directly. This optimized HMM lies at the core of the test system developed to assess the performance of parametric methods. The chimeric, artificial genomes provide a level playing ground for parametric methods to perform upon and be evaluated, i.e., we expect methods that perform well in detecting atypical genes in artificial genomes to perform well in classifying the genes in genuine genomes.

Comparative assessment of the parametric methods using the test system that we developed provides several insights. We observed that Karlin's dinucleotide method was outperformed by methods that used codon bias as a discriminant measure (see Figure 5B). However, we also found that a frame-specific dinucleotide measure implemented in an AIC-based clustering algorithm better discriminated the native and foreign genes than did codon bias measures implemented by any other algorithm. Therefore, the performance of a method depends both on the choice of statistic and on the methodology used. Methods like *k*-means clustering showed a significant variation in performance with the number of donor genomes (see Tables 1 and 2), and setting *k* = 2 does not seem to be a suitable choice for discriminating the pool of foreign genes from the native one. The donor genes originating from one source genome have distinct variability with respect to other genes, so the two-cluster approach may not always be a viable choice; increasing *k* can allow the method to create more centers for the genes to cluster around according to the genotypic variability inherent in a genome. Indeed, we have seen that an HMM with multiple gene models derived from gene clusters using the *k*-means method generates an artificial genome having characteristic variations of its genuine counterpart.

### Other Approaches for Deducing Gene Ancestry

In theory, comparing an organism's gene inventory to that of a close relative would provide one measure as to which genes were native (those shared between the two genomes) and which genes were foreign (those unique to the genome of interest). This approach has been applied to analyses of foreign gene detection with some success [13]. This phylogenetic approach has several weaknesses, which can color attempts to tune the performance of methods for atypical gene detection or to validate the analysis of any one genome sequence. First, there are many organisms for which no close relatives have been sequenced; in these cases, there are no suitable genomes to provide a basis for comparison. Second, the presence of a gene only in the taxon of interest may result from gain in that lineage or from loss in the sister lineage; the polarity of this event can be determined only by the analysis of three or more genomes. Third, there is a large degree of variability in gene content even among very closely related taxa—e.g., strains of *E. coli* share less than half of their species-wide gene inventories [29,39]—which will confound the identification of lineage-specific genes.

Last, and most important, genes shared among two genomes are “native” only from the perspective that they were present in the common ancestor of those two strains. That is, one would arrive upon very different inventories of “foreign” genes if the *Salmonella typhimurium* genome were compared to the *Salmonella typhi* genome, the *E. coli* genome, or the *Yersinia pestis* genome. To validate and calibrate parametric methods for detecting laterally transferred genes, assignment of genes as being “foreign” or “native” should not rely upon the designation of a particular outgroup taxon.

### Combining Approaches for Detecting Foreign Genes

Different sets of putative foreign genes are identified by different parametric methods in genuine genomes [9,10], leading to the conjecture that different methods detect different subsets of foreign genes. We believe that this hypothesis is supported by our finding that different methods for detecting foreign genes performed noticeably different in detecting genes from different sources (see Table 1). Because the identities of foreign genes are known with certainty in artificial genomes, we could test the hypothesis that a combination of methods that performed differently could, in tandem, outperform each method when used alone. Two strategies could then be implemented. One option is to relax discriminant criteria for the methods of atypical gene detection, thus identifying more foreign genes, but at the expense of misclassifying more native genes as potentially foreign (see Figure 5). The final set of putative foreign genes would be defined as those genes identified by all methods (the intersection of all gene sets). We do not favor this approach, because each method has difficulty in identifying particular foreign genes, and one would not expect them to appear in all sets.

Alternatively, one could use more stringent threshold criteria for atypical gene detection, thus misclassifying fewer native genes and minimizing type II error. The final set of putative foreign genes would comprise all atypical genes detected (the union of all gene sets). We favor this approach, because one method should identify some foreign genes that are not identified by the other. In addition, analysis of error rates (see Figure 5) allows us to choose threshold criteria that are conservative for each method. To this end, we identified putative foreign genes in chimeric artificial genomes using two of Karlin's methods, those using DNC and CUB as discriminant criteria. These two methods showed complementary strengths and weaknesses in identifying genes from different donor genomes (see Table 1).

To combine results, we selected threshold criteria that were more conservative than the optimal values, i.e., fewer native genes were misclassified as foreign at the expense of fewer foreign genes being correctly identified. However, when the results of the two methods were combined—i.e., we declared as foreign any gene that was so identified by either method—then the results of the combined methodology outperformed either method alone (Table 3). The mean error rate of the combined method (22.9%) was also less than the mean error rate of the component methods at their respective optimal thresholds (37.7% and 26.1% for Karlin's dinucleotide and codon bias methods, respectively). Therefore, we believe the artificial genome platform has justified the concept of a combined foreign gene identification approach whereby the union of sets of genes identified by different methods is denoted as “foreign.” We believe the strong improvement in detecting atypical genes reflects a “complementarity” of the methods, i.e., atypical genes detected well by one method were not detected well by the other, and vice versa. The three AIC-based methods showed less complementarity (see Table 1). When these methods were used together, the most prominent improvement in performance was observed for the combination of the AIC nucleotide and AIC codon bias methods (mean error rate of 13.8 compared to 14.9 and 15.2 at optimal thresholds for the AIC nucleotide and AIC codon bias methods, respectively; Tables 1 and S1). Understandably, addition of the AIC dinucleotide method yielded no additional improvements (Table S1), likely because this method does not augment detection of classes of genes left undetected by the combination of the other two methods. A notable feature of this analysis is that in all cases the type I error decreased by a large margin while the mean error rate remained almost same or less than those of the component methods at optimal thresholds (Tables 3 and S1). The combination of methods is thus suited for increasing the sensitivity substantially while keeping the number of false-positive results to a minimum.

**Table 3:**
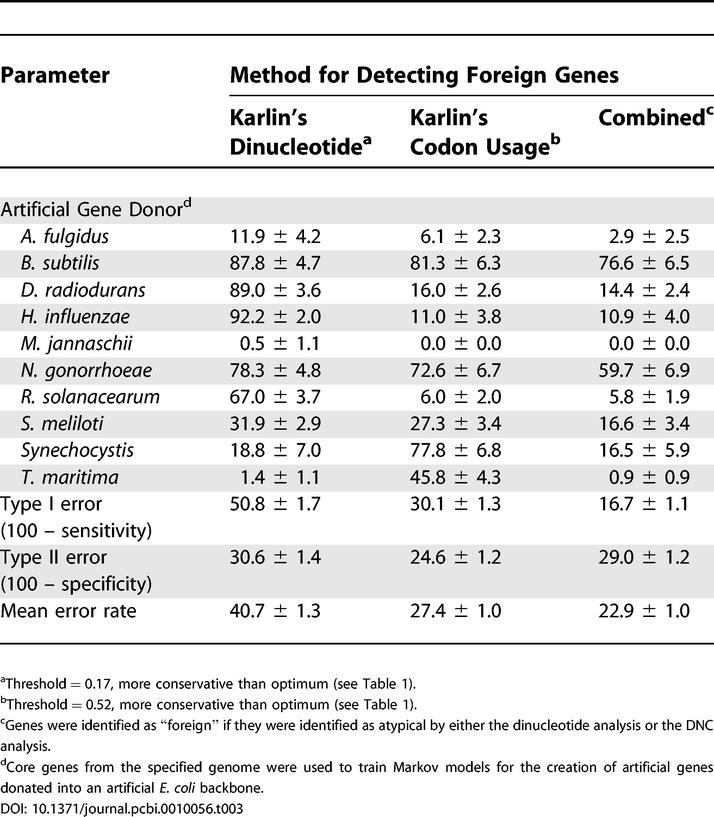
Performance of Combined Parametric Methods for Detecting Atypical Genes in Prokaryotic Genomes

### A Novel Method for Detecting Foreign Genes

Novel gene clustering algorithms based on the AIC [20] have also been proposed. These methods use the AIC to cluster genes by any parametric measure (e.g., DNC). These methods compared favorably with the existing parametric methods of atypical gene detection, clearly outperforming them in our test sets (see Tables 1 and 2; Figure 5B). Whereas the *k*-means clustering algorithm selects an arbitrary number of clusters *(k)* into which genes are apportioned, the AIC-based clustering algorithm segregates the genes into distinct gene classes reflecting the inherent complexity underlying the given genome. Unlike the current parametric methods, which merely detect unusual genes, it has the ability to distinguish between distinct classes of acquired genes, i.e., it identifies sets of genes that are atypical in a particular way. This property may be useful in identifying genes that were acquired from similar sources and thus bear similar sequence signatures. In addition, this feature may serve as a validation technique, where operons of foreign genes would comprise genes that fall into the same AIC-defined clusters.

The performance of the AIC-based methods was not influenced by use of the AIC in the method for identifying “core” genes for use in training Markov models to generate artificial genomes. To ensure the independence of these methods, we extracted the core genome using a method based on K-L distance (see Materials and Methods). The core of the *E. coli* genome selected by the K-L method contained 2,445 genes, where 1,788 of the genes were shared with the AIC-generated core. Because the core genomes produced by the two methods contain many of the same genes, the method used to select the core does not appear to bias the composition of the core. Rather, the differences reflect the relative stringency of the selection method. When methods for detecting atypical genes were assessed using chimeric genomes created using models of these core genomes, no significant differences were detected (see Figure S3; compare to Figure 5B). These results support the hypothesis that little, if any, bias remains in the composition of the core genome, and any bias would have been eliminated upon the creation of a chimeric artificial genome from genes created by several hundred Markov models. Therefore, we conclude that this approach provides a robust platform for evaluating the performance of parametric methods for the detection of atypical genes in bacterial genomes.

### Conclusions

Identifying the atypical character of a gene is a first step in identifying and quantifying lateral gene transfer events. Even though parametric methods have proved to be very effective in classifying foreign genes, reducing the margin of error is still a challenge. Our probabilistic approach is a step forward in assessing the atypical nature of genes through parametric methods using different null hypotheses and provides a platform for developing an integrated system of approaches that can assign a confidence value to a gene to be called typical or atypical, thus opening a new direction in quantifying lateral gene flow. Use of the HMM allows for artificial chimeric genomes to be generated given any set of prokaryotic genomes. This provides an objective test bed for evaluating the performance of newly proposed methods for atypical gene detection.

## Materials and Methods

### 

#### Genomes.

The complete genome sequences of several prokaryotic organisms—*A. fulgidus* DSM4304, *B. subtilis* 168, *D. radiodurans* R1 chromosome I, *E. coli* K12, *H. influenzae* Rd KW20, *M. jannaschii* DSM2661, *N. gonorrhoeae* FA1090, *R. solanacearum* GMI1000, *S. meliloti* 1021, *Synechocystis* sp. PCC6803, and *T. maritime* MSB8—were retrieved from GenBank. Open reading frames were extracted using coordinates provided in the annotation; for assigning genes to leading and lagging strands, the origins and termini of replication were localized using cumulative nucleotide skew [40,41].

#### Generalized HMM as a descriptor of a genome sequence.

Markov models have been successfully applied in deciphering the complex structural and functional units of a genome [42]. Borodovsky et al. [43] provided a rigorous mathematical framework in the form of a codon position−specific inhomogeneous Markov model for describing protein-coding sequences and a homogeneous Markov model for describing noncoding sequences. Markov models have been used extensively in gene-finding algorithms applied to both prokaryotic and eukaryotic genomes [19,44−47]. At the heart of such algorithms is an HMM incorporating models of distinct sequence types. The problem is formulated as deciphering the sequence of “hidden” states (e.g., protein-coding or noncoding) underlying a DNA sequence.

**Figure 7:**
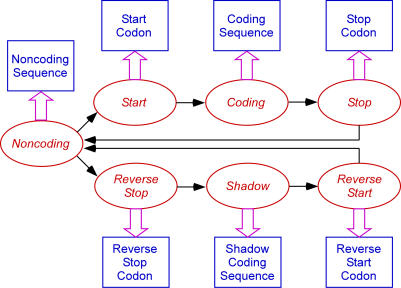
The HMM Architecture The oval represents a hidden state, and the square represents observation sequence. Each state emits a string of nucleotides and then makes a transition to another state. The transitions that are allowed between hidden states are shown by line arrows, and the emission of observation sequence is shown by block arrows. This HMM generates one strand of a genome; by including models of reverse complement of protein-coding sequence (“Shadow”), the HMM encapsulates the information of sequences on both strands of DNA helix. The “Reverse Start” and “Reverse Stop” states correspond to the reverse complement of start codons and stop codons, respectively.

Given the model parameters, a generalized HMM can be used to either predict desired features in a test sequence (e.g., find sequences that resemble genes) or generate a DNA sequence (e.g., create sequences that resemble genes). A simple, generalized HMM (Figure 7) can generate a genome sequence by choosing an oligonucleotide 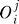 (*O* ∈ 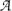, 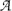 = {A,T,C,G}, where *i* and *j* indicate the start and end positions of the oligonucleotide in DNA sequence, respectively) according to output probability distribution *Q* in state *S_i_*. The length of the emitted sequence is determined by the length distribution of the sequences of a distinct state type (e.g.*,* the length of genes or the length of noncoding sequences). Transitions from state *S_i_* to *S_k_* (e.g., from a protein-coding state to a noncoding state) are made according to the probability distribution of transitions between states; this process is repeated until a genome sequence of a desired length *L* is generated.

Training sets were derived using the annotations provided in the GenBank sequences, and model parameters were obtained as the maximum likelihood estimate. For a gene model, the initial probability *P^ i^*(*O*_1_ *^m^*) of observing an oligonucleotide *O*_1_ *^m^* is estimated as


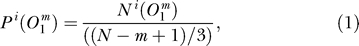


where *N^ i^*(*O*_1_ *^m^*) is the number of occurrences of oligonucleotide *O*_1_ *^m^* in phase *i* in the training data (phase *i* corresponds to the *i*th-codon position of the first base of the oligonucleotide). *N – m +* 1 is the count of all possible oligonucleotides of size *m* in the training data_._ The maximum likelihood estimate of the transition probability estimate 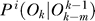 is given by


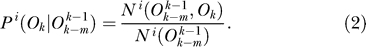


Here, 
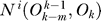
 and 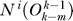 are the counts of the oligonucleotides 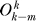 and 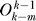 , respectively, in phase *i.* For noncoding sequences, the phase consideration is omitted. The values of all other probabilistic parameters (including the state initial and transition probabilities) were obtained as maximum likelihood estimates from the training set. The distribution of the number of genes in the same orientation, as well as the length distribution of protein-coding and noncoding sequences, was estimated from the GenBank annotation.

An HMM with a single gene model will fail to represent the variability of genes within a genome. There are distinct classes of genes that have evolved under different selective constraints, including genes encoded on leading and lagging strands, and variation in selection on codon usage, which requires the HMM to have multiple gene models. The HMM used for the creation of artificial genomes includes separate, multiple models of protein-coding sequence (its reverse complement or the protein-coding shadow) on both the leading and lagging strands, a model of noncoding sequence, and a model of gene orientation (see Figure S4).

#### Gene clustering by a *k*-means algorithm using K-L divergence.

To build multiple gene models, genes were segregated into distinct gene classes signifying divergent mutational biases. We adapted the *k*-means gene clustering method suggested by Hayes and Borodovsky [22] to perform this task. The (dis)similarity of two genes can be quantified in terms of their nucleotide composition or codon usage pattern. To quantify the difference in nucleotide composition between two genes or clusters of genes, *F* and *Q,* the difference is defined in the symmetric form of K-L divergence [22] as


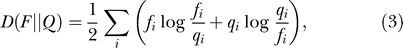


where *f_i_* and *q_i_* denote the relative frequencies quantitating the nucleotide patterns of the DNA sequences of genes *F* and *Q,* respectively. To quantify the difference in codon usage pattern, *D* is defined as


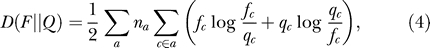


where *f_c_* and *q_c_* are the codon frequencies, *c,* normalized in the *a*th group of synonymous codons to which it belongs, for *F* and *Q,* respectively; and *n_a_* is the size of the *a*th group of synonymous codons. Note that for a cluster of genes, the center of the cluster is represented by the cumulative frequencies normalized in the respective groups.

The *k*-means gene clustering algorithm was initialized by selecting open reading frame cluster seeds by distributing genes at random among *k* clusters and calculating the cluster centers. Genes were reassigned to the cluster with the closest cluster center (in terms of *D*[*F||Q*] distance), and cluster centers were recalculated, until all genes resided within clusters with the closest centers. This process was repeated for several random realizations of the cluster seeds to eliminate any bias due to initial cluster assignment; the gene cluster configuration that minimizes the distance function Ψ,


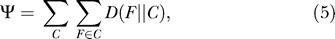


where *C* denotes cluster of genes, was selected.

#### Methods to extract the core genes of a genome.

We implemented two alternative approaches to extract the genes forming the “core” genome, those genes experiencing the range of naturally imparted mutational biases. First, genes were sorted into approximately 25 clusters by the *k*-means clustering algorithm using K-L divergence as the distance measure between clusters (see above). The two clusters whose centers were closest to each other were merged, and this process was repeated until the relative change in the K-L distance, *R,* between closest clusters exceeds the established threshold of


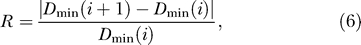


where *D*_min_(*i*) is the K-L distance between the two closest clusters at the *i*th iteration. The largest cluster was retrieved for each of the three discriminant criteria—nucleotide composition, DNC, and CUB—and the genes common to these three sets were taken to represent the core.

A drawback of using the K-L distance measure for finding gene clusters is that one begins with a arbitrary number of gene clusters that are then merged; therefore, the members of the final genome core will be biased both by the composition of the initial clusters and by the degree of variation within a genome. We sought to eliminate this bias by introducing a more rigorous method for cluster formation that did not begin by arbitrarily assigning genes to a fixed number of clusters. Rather, we sought to merge genes into clusters if they were similar to other members of that cluster. Criteria that can be used to select between models include the AIC [20], the minimum description length [48], and the Bayesian information criterion [49]. These approaches are based on the principle of finding the most parsimonious model, thus avoiding underfitting or overfitting models. After testing each criterion of model selection, we converged on the AIC for identification of core genes, which performed well and showed no bias with respect to cluster size or composition. The AIC is defined as


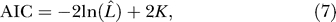


where 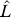 is the maximum likelihood and *K* is the number of free parameters in the model; the best-fitting model minimizes the AIC.

We used the AIC to determine if a one-gene (cluster) model significantly improves upon a two-gene (cluster) model. Among all possible pairings of gene clusters, the one minimizing reduction in likelihood of the gene cluster set was selected and this process was repeated to segregate genes into distinct clusters. The AIC provided the stopping criterion for the clustering procedure. In practice, for *N* genes, *N* single gene clusters were examined, and the pair of clusters with the least likelihood decrease were merged, resulting in *N –* 1 clusters. This process was repeated until the AIC for the merged cluster model was no longer less than the AIC for the separate cluster model. The largest cluster was retrieved for each of the three discriminant criteria—SNC, DNC, and CUB—and the genes common to these three sets were taken to represent the core genome.

To account for base compositional bias, the likelihood function 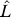 can be expressed in terms of frequencies of nucleotides (or oligonucleotides) located at specific codon positions. Considering single nucleotide statistics, a 12-dimensional frequency vector with frequencies of elements *b* ∈ {A*_i _,* T*_i _,* C*_i _,* G*_i_*}, *i* = 1, 2, 3, which takes into account both base identity and codon position, was used to calculate the maximum likelihood 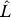; the likelihood for the separate cluster model was obtained as


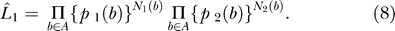


The likelihood for the merged cluster model was obtained as


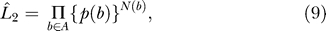


where {*p*(*b*)} and {*p*_1_(*b*), *p*_2_(*b*)} are the probabilities of base *b* in merged cluster and the two component clusters, respectively. *N*(*b*) denotes the count of base *b* in respective clusters. To allow the merger of the two clusters, the AIC of the merged cluster model should be less than the AIC of the two-cluster model. We assessed this difference as


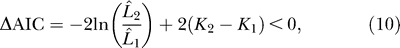


where *K*_1_ and *K*_2_ are the number of free parameters and 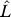
_1_ and 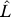
_2_ are the corresponding likelihoods in the two random cluster model and one random cluster model, respectively. The likelihood function, and thus the stopping criterion, can be obtained similarly for oligonucleotide statistics and also for CUB consideration.

#### Methods for detection of atypical genes.

Several widely used, parametric methods used in lateral gene transfer detection were implemented as follows. Karlin's dinucleotide bias [32] was assessed through use of the odds ratio:


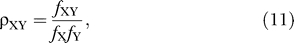


where *f*_XY_ is the frequency of the dinucleotide XY and *f*_X_ is the frequency of the nucleotide X. The dinucleotide average relative abundance difference between two DNA sequences *f* and *g* is defined as


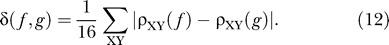


If the value of *δ* for a gene compared to average over all genes in a genome is greater than an established threshold, the gene is classified as foreign. Karlin's codon usage difference [36] of the gene family *F* relative to gene family *C* was quantified as


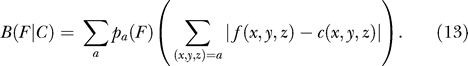


where {*f*(*x,y,z*)} is the set of codon frequencies for the gene family *F,* {*c*(*x,y,z*)} is the set of codon frequencies for the gene family *C,* and {*p_a_*(*F*)} is the set of amino acid frequencies of the genes of *F*.

The codon frequencies were normalized to one in each amino acid codon family, so that


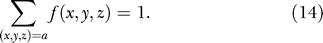


If *C* is the set of all genes and *F* is a single gene, *B*(*F*|*C*) = *B*(*F*|all) measures the codon bias of *F* compared to the average for all genes. If *B*(*F*|all) is greater than an established threshold, *F* is classified as a foreign gene.

CUB was used as a discriminant criterion in the *k*-means gene clustering algorithm of Hayes and Borodovsky [22], where relative entropy was used as a distance measure of codon usage difference between clusters of genes (see equation 4) in a *k*-means algorithm. We also implemented base compositional bias and CUB as discriminant criteria in an AIC-based gene clustering algorithm. We have already discussed the utility of the AIC in identifying the genes likely to be forming the native core of a genome. We also tested the performance of an AIC-based gene clustering algorithm in identifying atypical genes. Note that we used a generalized version of the AIC, defined as


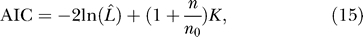


where *n* is the sample size and *n_0_* is a positive constant [50]. For *n*_0_ *= n,* the generalized version takes the form of a standard AIC (see equation 7). The tuning parameter *n*_0_ was used to optimize the algorithm.

## Supporting Information

Figure S1 Correspondence Analysis of CUB The first axes—indicating variability in usage among 59 synonymous codons—are plotted for 4,255 *E. coli* genes (A), 2,141 *E. coli* genes representing the “core” genome (B), and 2,141 genes comprising the artificial *E. coli* core genome (C). The artificial genome was created from genes clustered by frame-specific DNC.
(826 KB TIF)

Figure S2 Variability within Genuine and Artificial *E. coli* Genomes The percent GC of third-codon positions is plotted for 4,255 *E. coli* genes (A), 2,141 “core” *E. coli* genes (B), and 2,141 genes within the artificial “core” *E. coli* genome (C). The artificial core genome was created from genes clustered by frame-specific DNC; μ and σ represent the mean and standard deviation of the distribution.
(388 KB TIF)

Figure S3 Tradeoffs in Error Rates for Several Methods of Gene Detection Artificial genomes were generated from Markov models trained on core genomes extracted using a K-L distance method. Compare to Figure 5B.
(142 KB TIF)

Figure S4 Cumulative GC-Skew Plots for Genuine and Artificial *E. coli* Genomes For each gene, GC skew was calculated as (%G – %C)/(%G + %C) of third-codon positions, corrected for direction of transcription. Beginning with the first gene in a genome sequence, cumulative skew was obtained as the sum of skew values for the preceding genes. The genuine *E. coli* genome comprised 4,255 protein-coding genes, whereas the artificial genomes comprised 4,000 genes. Cumulative GC-skew plots are shown for artificial *E. coli* genomes with and without gene models accounting for strand bias during training. Strand bias is evident as large domains of either G-rich or C-rich genes in the genuine *E. coli* genome and in the artificial genome with strand identity incorporated into the model.
(273 KB TIF)

Table S1 Performance of Combined Parametric Methods for Detecting Atypical Genes in Prokaryotic Genomes (42 KB DOC)

## Abbreviations

AIC: Akaike information criterion
CUB: codon usage bias
DNC: dinucleotide composition
HMM: hidden Markov model
K-L: Kullback-Leibler
SNC: single nucleotide composition
