# Supplementary material for: Use of Artificial Genomes in Assessing Methods for Atypical Gene Detection
Source: PLoS Comput Biol. 2005 Nov 11;1(6):e56. doi: 10.1371/journal.pcbi.0010056 (PMC1282332; doi:10.1371/journal.pcbi.0010056)
Supplement: Supplementary file 1 [file pcbi.0010056.st001.doc]

**Supplementary Table 1**. Performance of combined parametric methods for detecting atypical genes in prokaryotic genomes

| Artificial Gene Donor | Error rate in classifying native or alien genes | | | | | |
| --- | --- | --- | --- | --- | --- | --- |
|  | AIC nuc ^2^ | AIC Dinuc ^3^ | AIC Codon ^4^ | AIC nuc &  AIC dinuc^5^ | AIC nuc &  AIC Codon^5^ | AIC nuc, AIC Dinuc  & AIC Codon^5^ |
| *A. fulgidus* | 49.8 ± 36.2 | 25.2 ± 42.1 | 2.4 ± 2.2 | 8.9 ± 20.6 | 1.4 ± 2.0 | 0.4 ± 1.1 |
| *B. subtilus* | 68.2 ± 17.5 | 79.5 ± 12.0 | 82.5 ± 11.0 | 59.1 ± 15.0 | 58.8 ± 18.5 | 51.8 ± 15.6 |
| *D. radiodurans* | 3.5 ± 2.7 | 2.8 ± 2.8 | 12.8 ± 29.1 | 2.3 ± 2.7 | 1.4 ± 1.5 | 1.0 ± 1.5 |
| *H. influenzae* | 2.9 ± 2.6 | 1.6 ± 1.3 | 1.9 ± 1.5 | 0.9 ± 0.9 | 0.5 ± 0.6 | 0.2 ± 0.5 |
| *M. jannaschii* | 0.1 ± 0.4 | 0.1 ± 0.5 | 0.7 ± 1.1 | 0.0 ± 0.0 | 0.0 ± 0.0 | 0.0 ± 0.0 |
| *N. gonorrheae* | 47.8 ± 14.2 | 77.1 ± 23.6 | 72.9 ± 21.4 | 42.8 ± 15.3 | 37.5 ± 8.7 | 35.7 ± 14.3 |
| *R. solanacaerum* | 0.3 ± 0.8 | 0.6 ± 0.8 | 13.9 ± 28.5 | 0.1 ± 0.4 | 0.3 ± 0.7 | 0.1 ± 0.4 |
| *S. meliloti* | 3.6 ±1.1 | 2.9 ± 2.0 | 16.7 ± 27.4 | 2.2 ± 1.5 | 1.6 ± 1.2 | 1.1 ± 1.1 |
| *Synechocystis* | 95.7 ±3.1 | 88.2 ± 19.2 | 42.4 ± 31.7 | 85.2 ± 18.4 | 37.0 ± 30.3 | 38.2 ± 30.1 |
| *T. maritima* | 44.4 ± 37.7 | 25.1 ± 42.9 | 6.4 ± 3.9 | 7.1 ± 20.9 | 2.8 ± 3.5 | 0.6 ± 1.4 |
|  |  |  |  |  |  |  |
| Error I  (100-sensitivity) | 24.9 ± 8.6 | 22.5 ± 10.6 | 20.4 ± 13.1 | 14.9 ± 5.4 | 10.0 ± 4.1 | 8.9 ± 3.7 |
| Error II  (100-specificity) | 8.2 ± 2.2 | 3.6 ± 3.0 | 13.2 ± 10.6 | 10.0 ± 2.4 | 17.7 ± 9.8 | 19.4 ± 9.0 |
| Mean Error Rate | 16.5 ± 3.6 | 13.1 ± 4.7 | 16.8 ± 6.2 | 12.5 ± 2.7 | 13.8 ± 4.1 | 14.2 ± 3.9 |

1. Core genes from the specified genome were used to train Markov models for the creation of artificial genes donated into an artificial *E. coli* backbone.

2. Threshold = 0.6, more conservative than optimum (See table 1).

3. Threshold = 2.0, more conservative than optimum (See table 1).

4. Threshold = 1.7, more conservative than optimum (See table 1).

5. Genes were identified as ‘alien’ if either they were identified as alien by any of the methods
